# Bacterial Chondronecrosis with Osteomyelitis (BCO) in Broiler Chickens: Bacterial Causes, Pathophysiology and Control, with a Focus on the Potential of Electron Beam-Inactivated Vaccines

**DOI:** 10.3390/vaccines14080649

**Published:** 2026-07-23

**Authors:** Udara Rathnayake, Ruvindu Perera, Palmy R. R. Jesudhasan, Adnan Alrubaye

**Affiliations:** 1 Cell and Molecular Biology Program, University of Arkansas, Fayetteville, AR 72701, USA; urathnayake@uark.edu (U.R.); rperera@uark.edu (R.P.); 2 Poultry Production and Product Safety Research Unit, United States Department of Agriculture—Agricultural Research Service, Fayetteville, AR 72701, USA; 3 Division of Agriculture, Center of Excellence for Poultry Science, University of Arkansas System, Fayetteville, AR 72701, USA

**Keywords:** chondronecrosis, osteomyelitis, eBeam, vaccine, antimicrobial resistance, broiler welfare, lameness

## Abstract

Bacterial chondronecrosis with osteomyelitis (BCO) is a major cause of lameness in modern broiler chickens. It remains a persistent challenge in the broiler industry, resulting in substantial economic losses and serious animal health concerns. The pathogenesis of BCO involves rapid muscle growth, resulting in disproportionate body weight relative to skeletal maturity, thereby increasing mechanical stress and leading to microfractures and osteochondrotic clefts in the proximal growth plates of the femora and tibiae. Infection progresses via hematogenous dissemination and colonization of the leg bones by opportunistic pathogens, primarily *Staphylococcus* spp., *Enterococcus* spp., and *Escherichia coli*, originating from the gastrointestinal or respiratory tract, leading to chronic inflammation, ischemia, biofilm formation, and progressive bone necrosis. Control strategies, including selective breeding, enhanced management and production practices, nutritional interventions, and the administration of antimicrobial agents, offer partial mitigation but have failed to provide a permanent solution to BCO incidence. Vaccination is one of the most promising and targeted immunological approaches to enhance the host immune responses against bacterial pathogens. Electron beam (eBeam) inactivated vaccines represent a significant advancement among available vaccination strategies. This review leverages current knowledge of the etiology, pathophysiology, control measures, and the use of eBeam technology (EBT) in vaccine development to provide a scientifically grounded and innovative approach to controlling BCO-associated lameness in broiler chickens. This article also provides insights into future directions for integrated, antibiotic-free strategies to mitigate BCO and revenue losses, enhance broiler welfare, ensure consumer safety, and support the long-term sustainability of the global poultry industry.

## 1. Introduction

The poultry industry is one of the most significant and commercially valuable sectors in the United States agricultural production [1]. The commercial broiler production process has improved through selective breeding [2,3,4]. Selective breeding has favored broiler genetic lines with rapid growth rates, reaching market weight in shorter periods [5,6]. This process impairs the proper development and functional maturation of internal organs [6,7,8,9,10]. The rapid increase in body weight (BW) in modern broilers often outpaces the growth and structural integrity of the skeletal system. As a result of this imbalance, modern broiler lines are susceptible to numerous health issues, and within the poultry industry, lameness is a prevalent case [11,12,13,14]. Bacterial chondronecrosis with osteomyelitis (BCO), previously known as femoral head necrosis, is one of the most predominant infectious musculoskeletal disorders or causes of lameness in modern broilers [13,15,16]. BCO lameness primarily affects the proximal femoral and tibial heads (growth plates and adjacent metaphyses). This condition often leads to bacterial infection, inflammation, and necrosis [17]. The most predominant causative agents of BCO lameness are *Staphylococcus* spp., *Escherichia coli*, and *Enterococcus* spp. [17,18]. BCO lameness causes major economic losses in the poultry industry, ranging from $900 million to $1.8 billion annually in the United States [18]. While typical BCO incidents result in flock losses ranging from 1 to 5% [17], episodic BCO outbreaks may reach up to 30% [12,15,19,20,21] of flocks infected. Several studies have focused on the efficient use of genetic selection, nutritional interventions, improved management strategies, and antimicrobials to reduce the incidence of BCO lameness in broiler chickens [22,23,24,25,26,27]. These approaches can partially delay disease development but are not successful in preventing BCO lameness in broiler chickens. Vaccination offers a targeted, sustainable approach to controlling BCO by stimulating specific immunity against key bacterial pathogens before they can establish infection in the bone [28]. Among emerging vaccine technologies, electron beam (eBeam) technology is noteworthy [29,30,31,32]. This review comprehensively examines the etiology, pathogenesis, pathophysiology, and current control measures for BCO lameness in broilers. It critically evaluates and compares the potential of bacterial vaccines, with special focus on the development, mechanism, and efficacy of eBeam-inactivated vaccines. The objective of this review is to highlight future perspectives on integrated, antibiotic-free strategies to reduce BCO incidences, improve broiler welfare, and strengthen the long-term sustainability of global poultry production.

## 2. Materials and Methods

The literature analysis in this review was conducted using the Google Scholar database as the primary source, supplemented by manual searches of reference lists from key articles. Search terms included combinations of the following search queries: “bacterial chondronecrosis with osteomyelitis”, “poultry”, “osteomyelitis”, “hematogenous translocation”, “broiler chicken”, “vaccine”, “eBeam inactivated vaccine”, “antimicrobial resistance”, “bacterial vaccines”, “BCO lameness”, “electron Beam technology”, “BCO pathophysiology”, “BCO control strategies”, “bacterial translocation”, “*Staphylococcus*”, “*Escherichia coli*”, and “*Enterococcus*”. Additional studies were identified through complementary approaches in addition to the keyword-based literature search. Inclusion criteria: the analysis includes English-language peer-reviewed articles, book chapters, case reports, and conference proceedings published primarily between 1950 and 2026. Exclusion criteria: non-English publications and unpublished data were excluded from the study analysis.

## 3. Etiology, Pathogenesis, and Pathophysiology of BCO Lameness

### 3.1. Role of Rapid Growth and Mechanical Stress

BCO lameness is primarily driven by disproportionate growth dynamics observed in modern broiler chickens, in which rapid body mass gain outpaces skeletal development [17,22]. Average broiler chicks weighing 40 g at hatch can increase their body mass to over 4 kg, nearly a hundredfold growth within 8 weeks [9,22]. The growth of leg bones involves axial elongation through growth plates at the ends of the diaphyseal shaft and radial expansion due to dynamic remodeling processes of cortical bone [17,22]. During the first six weeks post-hatch, the femora and tibiae increase in length by approximately four times, while mid-shaft diameter increases between three and five times [33,34]. During the brief lifespan of modern broilers, rapid musculoskeletal development results in disproportionate body weight relative to skeletal maturity, increasing shear stress and torsional forces on developing juvenile leg bones during locomotion [17]. In broiler femora and tibiae, long chondrocyte columns in the proximal growth plates are highly susceptible to mechanical stress [22]. This stress induces microfractures between and within the cartilage layers, reducing blood circulation and ultimately leading to necrosis [35]. Proximal ends of the femora and tibiae are more prone to lesion development compared to the distal ends of the same bones due to longer columns of chondrocytes and thicker growth plates [18,36]. Infections of proximal femoral and tibial osteochondrotic clefts often result from bacterial translocation from the gastrointestinal (GIT) or respiratory (RT) tract or from environmental exposure, leading to progressive necrosis and associated lameness in broiler chickens [17]. However, the rapid growth rate is not the sole determinant of the risk of BCO lameness in broiler chickens [37].

### 3.2. Pathophysiology of BCO Lameness

**Figure 1:**
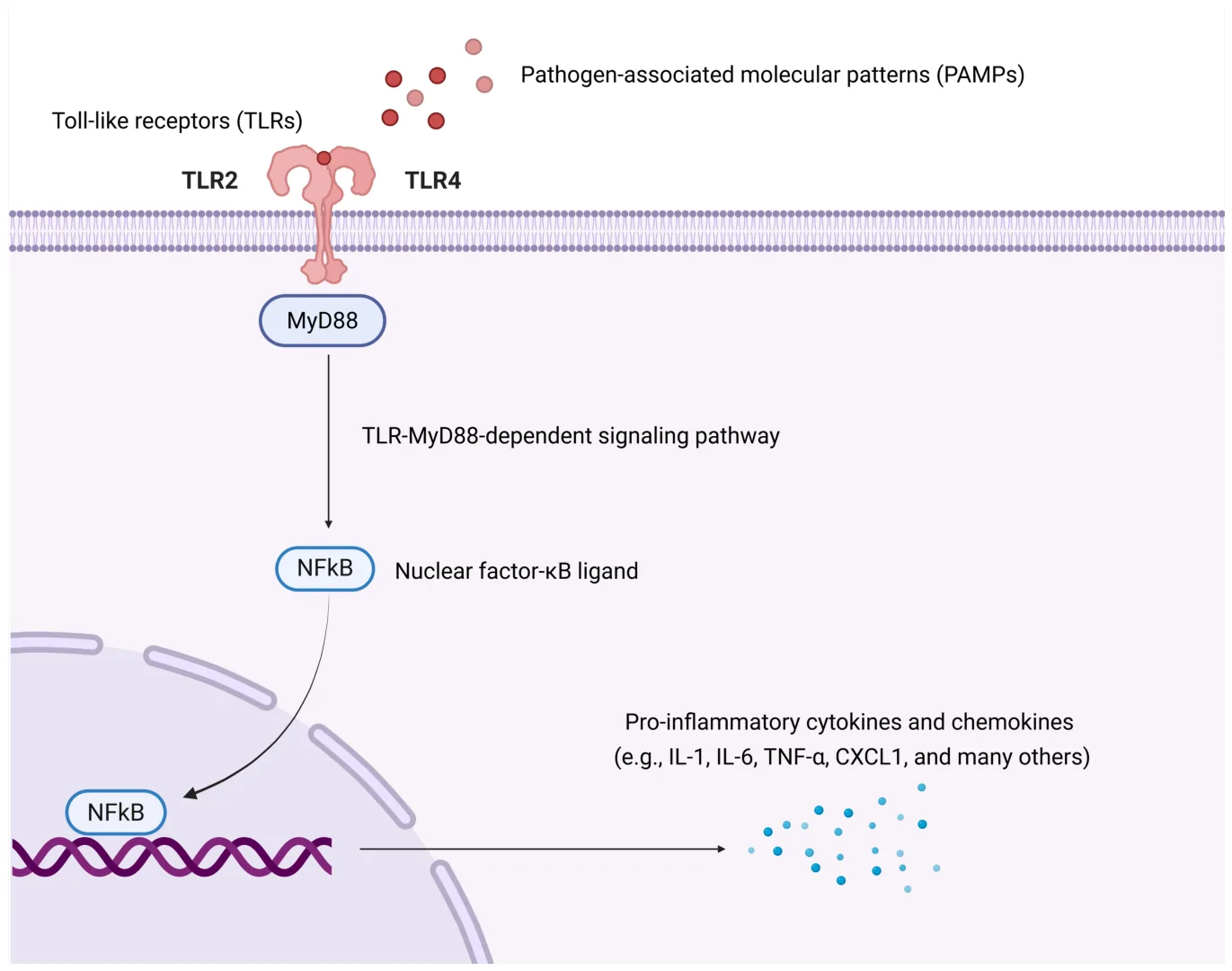
Schematic diagram of the TLR-MyD88-dependent signaling pathway. During bacterial invasions, resident osteocytes detect bacterial components. Pathogen-associated molecular patterns (PAMPs) bind to toll-like receptors (TLR2/TLR4) of the osteocyte cell wall, and this binding activates the TLR-MyD88-dependent signaling pathway. This association promotes receptor activator of nuclear factor-κB ligand (NFKB) expression, and secretes pro-inflammatory cytokines and chemokines (e.g., IL-1, IL-6, TNF-α, CXCL1, and many others) to recruit immune cells. Created in BioRender. Rathnayake. (2026) (https://biorender.com/).

The rapid growth promotes focal ischemia and provides a favorable environment for pathogen colonization [16,17,22]. The establishment of pathogens at the site of infection initiates intense local inflammatory responses. At this juncture, resident osteocytes initiate local immunity as sensory agents of mechanical stress within the bone microenvironment [38,39]. These osteocytes detect bacterial components such as peptidoglycan (PGN), lipopolysaccharide (LPS), and lipoteichoic acid at the site of infection through pattern recognition receptors (PRRs), including toll-like receptors (TLRs) [38]. TLRs detect conserved pathogen-associated molecular patterns (PAMPs) and damage-associated molecular patterns (DAMPs) [40]. Bacterial PAMPs bind to TLR2 or TLR4 on the osteocyte surface, and this binding activates the TLR-MyD88-dependent signaling pathway. This association promotes receptor activator of nuclear factor-κB (NFκB) ligand (RANKL) expression, and secretes pro-inflammatory cytokines and chemokines (e.g., IL-1, IL-6, TNF-α, CXCL1, and many others) to recruit immune cells [41] (Figure 1). Moreover, the TLR-MyD88-dependent signaling pathway stimulates osteoclast-mediated bone resorption and attracts macrophages and neutrophils to the site of infection [42]. The resulting acute inflammation creates an avascular environment that hampers immune clearance and ultimately leads to edema, vascular thrombosis, ischemia, and rapid tissue necrosis. In chronic osteomyelitis, pathogens may evade host defenses by infiltrating the sub-micron canaliculi of the osteocyte lacuno-canalicular network (OLCN) of cortical bone and forming biofilm-protected reservoirs that sustain persistent infection [43]. This protected reservoir acts as a physical barrier against host immune cells and antibiotic treatments [44,45].

### 3.3. Bacterial Causes and Translocation Mechanisms

BCO epiphyseal lesions are predominantly associated with bacteria of the genus *Staphylococcus.* At the same time, *Enterococcus* spp., *E. coli*, *Mycobacterium* spp., and *Salmonella* spp. have also been reported among the isolated bacteria [20,46,47,48]. Among *Staphylococcus* species, *S. aureus* has been strongly associated with BCO lesions and associated lameness [18]. However, *S. agnetis* has also been recognized as an emerging pathogen of interest due to the presence of distinctive virulence factors, which share homology with both pathogenic and non-pathogenic Staphylococci [18,49]. The bacterial invasion is associated with hematogenous translocation (HT) of opportunistic bacteria originating from the intestinal microbiota, respiratory tract, and integument [17,35,50]. Other potential sources of bacterial transmission to chicks include breeder parent stock, contaminated eggshells, or the hatchery environment [51,52]. Translocation of bacteria from the GIT due to impaired intestinal barrier integrity, often described as “leaky gut,” is a widely proposed mechanism for BCO pathogenesis [17,22,53]. Integrity and permeability of the epithelial cell layer lining the small intestine are regulated by tight junctions (TJ), which consist of multiprotein complexes such as occludin and claudin [54]. These TJs and the associated epithelial layer form the primary barrier between the small intestine and the bloodstream, which can be disrupted by enteric bacteria [53,54]. Other contributing factors, such as environmental stress, impaired immunity, or poor nutrition, can compromise TJ integrity and function, thereby increasing epithelial permeability and facilitating HT of pathogenic bacteria [17,22]. Moreover, in commercial production settings, including hatcheries and production houses, broiler chickens are frequently exposed to airborne pathogens. In immunocompromised or stressed birds, these inhaled bacteria can colonize the mucosal layer of the upper RT and the avian air sacs. Subsequent proliferation of these bacteria can lead to their translocation into the bloodstream, thereby contributing to BCO infections [18,46]. This exposure may lead to prolonged health issues and ultimately affect consumer food safety [55,56].

## 4. Non-Vaccine Control and Mitigatory Strategies for BCO Lameness

Table 1 provides an overview of non-vaccine early preventive and mitigation strategies for BCO lameness in broiler chickens, including their reported efficacy and limitations [57,58].

### 4.1. Genetic Selection and Breeding Approaches

Genetic selection has played a key role in improving growth performance, feed efficiency, and breast muscle yield, however, it has exacerbated skeletal challenges in modern broiler strains [86]. Selective breeding programs have incorporated skeletal robustness as a primary trait to balance growth performance and structural integrity. In modern broiler breeding programs, characters such as tibial breaking strength, cortical thickness, bone mineral density, and gait scores have been used as indicators of skeletal quality. Several studies have suggested that broiler lines with these improved traits have reduced the incidence of musculoskeletal abnormalities, including angular deformities, tibial dyschondroplasia, and lameness [60,61]. Genomic selection and quantitative trait analysis for identifying alleles associated with enhanced tibial strength, bone density, mineralization, and mechanical resilience have increased the ability to withstand rapid growth-related stress, thereby reducing microfracture formation and the risk of subsequent bacterial colonization and BCO infection [4,6,17,62]. Modern breeding programs strongly incorporate welfare indicators, including leg health, locomotion, and activity levels, rather than traditional production traits, to reduce skeletal disorders and improve overall broiler welfare [61].

### 4.2. Effective Management of the Broiler Production Environment

Lighting regimes (duration, spectrum, and intensity) in broiler production systems have been intensively studied to evaluate their effects on behavior, skeletal development, feed intake, and growth performance [65,87]. Broilers are commonly provided with 23 h of light (photoperiod) and 1 h of dark (scotoperiod) (23 L:1D) during the initial brooding period (typically for the first 7 days) to encourage early feed intake and growth [88]. During the grow-out period, 20L:4D or 18L:6D light periods are used. However, practices vary by region, integrator, and welfare concerns [89]. Recent research has shown that a shorter photoperiod and a longer scotoperiod may yield greater production benefits [90,91]. Additionally, exposure to blue and green light spectra promotes walking, wing flapping, and exploratory activity, thereby enhancing the mechanical stimulation of bone tissue [61]. Furthermore, balanced light intensities are crucial for broiler skeletal development and health, as excessive light intensity increases mechanical stress due to hyperactivity [92,93].

Effective regulation of ventilation ensures optimal environmental conditions by eliminating excess heat, moisture, dust, ammonia, and carbon dioxide, thereby supporting bird welfare, physiological health, and overall growth [94]. Accumulation of ammonia and carbon dioxide in poorly ventilated housing systems can cause respiratory distress, oxidative stress, and reduced feed intake, leading to impaired bone mineralization and development [69,70,95]. To maintain optimal air quality and consistent airflow, modern broiler houses commonly use tunnel or negative-pressure ventilation systems [61]. Recent research indicates that broilers reared under properly ventilated conditions showed higher tibial breaking strength, improved cortical thickness, and a lower rate of leg abnormalities [61].

Maintaining proper thermal conditions is also vital for broiler chickens, which are highly sensitive to temperature fluctuations due to their high metabolic rate, high body temperature, and rapid growth [96]. When ambient temperatures exceed 32 °C, the resulting heat stress can reduce feed intake, suppress thyroid hormone activity, impair osteoblast proliferation, limit calcium (Ca) and phosphorus (P) absorption, ultimately reducing bone mineralization [97,98,99]. Heat stress also promotes oxidative stress markers and suppresses the activity of antioxidant enzymes, thereby negatively affecting bone formation [100]. Conversely, cold stress increases the metabolic demands for thermoregulation [101]. Therefore, broiler production houses commonly use effective housing management strategies to maintain thermal balance and prevent stress-induced skeletal disorders [61,101,102].

### 4.3. Nutritional Interventions

Nutritional interventions remain a cornerstone of skeletal health in fast-growing broilers, as they play a remarkable role in bone growth, mineralization, structural adaptation, and integrity through optimal macro- and micro-nutrient intake and energy-protein provision [61]. Feed supplementation with probiotics, vitamins, trace minerals and amino acids are commonly used to mitigate BCO in broiler chickens [17,62,103]. The use of probiotics in diets has emerged as a promising approach in broiler nutrition due to their significant potential to reduce skeletal disorders in broiler chickens [104]. Several studies have shown that probiotics enhance intestinal integrity, immune function, leg health, and overall growth performance [47,105]. Trace minerals, including manganese (Mn), zinc (Zn), copper (Cu), and boron (B), are commonly supplemented in broiler diets to regulate immune function and support connective tissue and bone development [27]. Furthermore, these minerals act as enzyme cofactors involved in chondrocyte differentiation, osteoblast activity, and collagen cross-linking [26,106]. Protein and amino acids are fundamental sources of nutrients in the diets of fast-growing broilers. Balanced intake of essential amino acids and protein supports metabolic function and promotes homeostasis [107]. Vitamins are also considered vital components of food critical for broiler development, growth, and reproduction. In intensive poultry farming systems, vitamin D_3_ supplementation has become a general practice to mitigate musculoskeletal disorders [74,75]. Ca and P contribute significantly to the formation of hydroxyapatite, mineralization of bone, and the regulation of osteoblast and osteoclast activity [76].

### 4.4. Use of Antimicrobials and Emergence of Antimicrobial Resistance

Antimicrobials are commonly administrated for disease prevention, treatment, and growth promotion in poultry production. However, antibiotic growth promoters have been banned in several countries [80,108]. Antibiotic usage for the treatment of infections generally caused by *Staphylococcus*, *Salmonella*, *E. coli*, or *Clostridium* spp. is permitted in all large-scale commercial poultry-producing countries. Antimicrobials such as tetracyclines, fluoroquinolones, macrolides, sulfonamides, aminoglycosides, polypeptides, and beta-lactam antibiotics have been found to reduce the incidence of bacterial diseases, including BCO, in poultry [81]. Antimicrobial therapy for BCO should be initiated as quickly as possible due to its highly destructive nature, and antimicrobials can effectively treat most acute cases. Almost all antibiotics readily penetrate bone and articular tissue [79,109].

Antimicrobial resistance (AMR) is defined as the capacity of a bacterium to resist the inhibitory or killing activity of an antimicrobial compound [110]. In the poultry industry, AMR represents a critical global health concern [111]. Bacteria suppress antibiotic activity through enzymatic degradation, target-site alteration, efflux pumps, gene transfer, biofilm formation, reduced membrane permeability, and plasmid-mediated resistance [112]. Some bacteria produce enzymes that can inactivate antibiotics, such as beta-lactamases and aminoglycoside-modifying enzymes [113,114]. Certain bacteria, including *Escherichia coli* and *Salmonella* spp., can modify the structure of the target site where the antibiotic is supposed to bind [115]. Efflux pumps reduce intracellular antibiotic accumulation to non-lethal levels by extruding it from bacterial cells [112]. Several bacteria including *E. coli* and *Salmonella* spp. can transfer resistance genes, such as the mcr-1 gene, into recipient bacteria via plasmids, bacteriophages, and other genetic elements through transduction [116,117]. Furthermore, *Salmonella* spp. produce surface layers or biofilms as a protective covering against antimicrobial agents and disinfectants, making them difficult to eliminate and control [112]. Certain bacteria, including *E. coli*, reduce membrane permeability by downregulating porin expression [118,119]. Many *Staphylococcus* species exhibit resistance to a broad range of antibiotics, and AMR is increasing [14]. For these reasons, antimicrobial drugs are not suitable as a long-term solution for bacterial diseases such as BCO [14]. Antibiotic therapy cannot reverse the damage already caused to the musculoskeletal system, and the BCO is likely to reappear soon after antibiotic withdrawal [24].

## 5. Critical Perspectives on Bacterial Vaccine Platforms in the Context of Broiler Production

### 5.1. Bacterial Vaccines as an Alternative to Antibiotics

**Figure 2:**
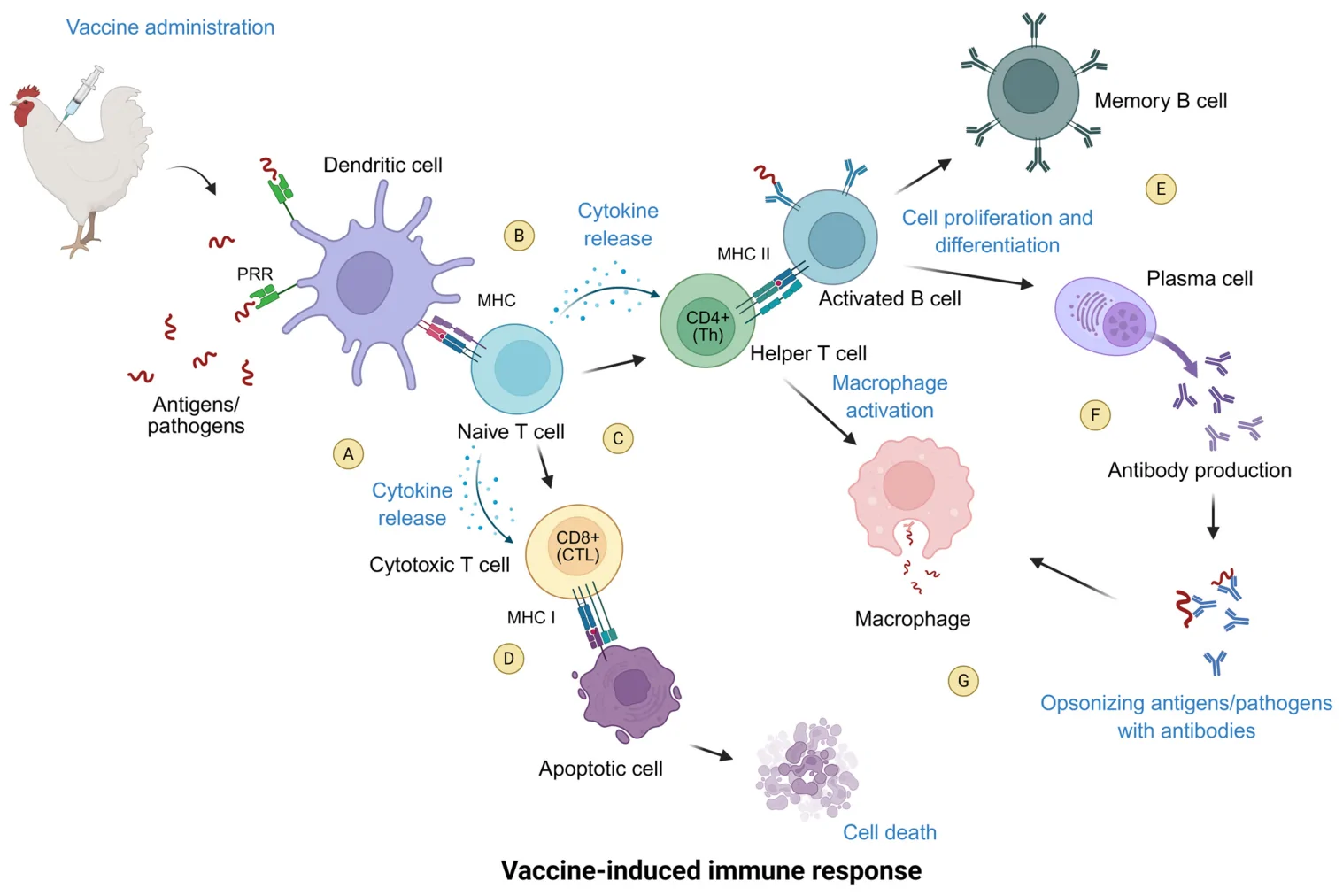
Schematic diagram of vaccine-induced immune response [130,131,132,133,134,135,136]. (**A**) Dendritic cells recognize vaccine antigens through pattern recognition receptors (PRRs). (**B**) Vaccine antigens are processed for antigen presentation on major histocompatibility complexes (MHCs) to naive T cells. Release cytokines that affect the differentiation of naive T cells. (**C**) Naive T cells differentiate into CD4+/helper T cells and CD8+/cytotoxic T cells. (**D**) Cytotoxic T cells kill infected host cells by triggering apoptosis (programmed cell death). (**E**) Antigen-specific activated B cells facilitate cell proliferation and differentiation into plasma cells or memory B cells. (**F**) Plasma cells produce antigen-specific antibodies that can opsonize pathogens. (**G**) Activate macrophages to recognize and phagocytose antibody-opsonized pathogens. Created in BioRender. Rathnayake. (2026) (https://biorender.com/).

Since the administration of antimicrobial agents for disease prevention and control is limited, alternative approaches such as bacterial vaccines are essential to prevent the emergence and spread of multidrug-resistant (MDR) bacteria carrying AMR in poultry [120,121,122]. Bacterial vaccines provide protective immunity against specific pathogens by mimicking infection, stimulating both innate and adaptive immune responses to offer targeted and sustained defense while reducing dependence on antibiotics [123] (Figure 2). When a vaccine is administered via oral, in ovo, spray, or injection routes, it is first recognized by the bird’s innate immune system. This promotes the production of key immune effectors, such as macrophages and neutrophils, which can recognize, engulf, and destroy pathogenic bacteria. These phagocytic cells can initiate inflammation and bridge to adaptive immunity by releasing cytokines and antimicrobial peptides. Vaccination can enhance innate immune defenses by inducing the production of specific antibodies against the antigens contained in the vaccine [83,124]. This trained immunity (TI) is mediated by prototypical innate immune cells, including monocytes, macrophages, and natural killer cells [125]. TI is primarily driven by two key pillars: epigenetic and metabolic reprogramming of cells [126,127]. Epigenetic reprogramming of innate immune cells induces TI against infections, while metabolic reprogramming increases their capacity to respond to secondary infections [128,129]. Bacterial vaccines integrate phagocytosis, humoral antibody production with memory B cell development, helper T cell activity, and cytotoxic T cell activity [130]. These mechanisms offer distinct strategies to prevent bacterial infections in broilers and provide a highly effective approach to replace antibiotic use.

### 5.2. Different Types of Bacterial Vaccines—A Critical Perspective

**Figure 3:**
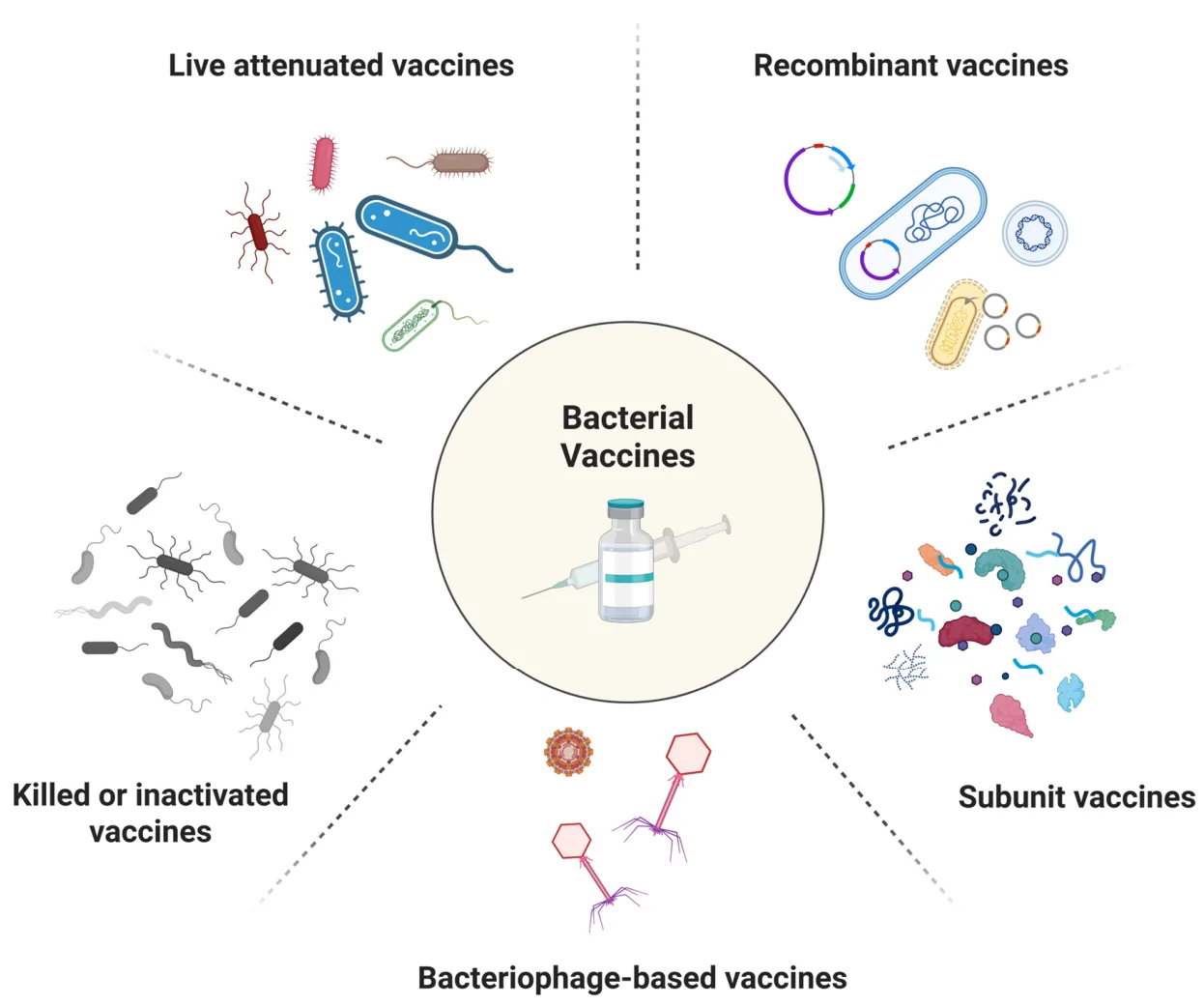
Different types of bacterial vaccines. Bacterial vaccines can be produced using different processes. Vaccines may contain live attenuated pathogens, inactivated whole pathogens (killed or inactivated), parts of pathogens (bacteriophage-based or subunit-based), or recombinant proteins/DNA. Created in BioRender. Rathnayake. (2026) (https://biorender.com/).

Bacterial vaccines are commonly classified into several major categories: killed or inactivated, live attenuated, recombinant, subunit, and bacteriophage-based vaccines [28] (Figure 3). But no single vaccine platform is universally considered superior; each type involves distinct immunological, practical, economic, and safety concerns [137].

Killed or inactivated bacterial vaccines are produced by culturing a specific pathogen in vitro and then eliminating its infectivity using chemical agents (e.g., formalin), heat, or physical methods (e.g., radiation) while preserving immunogenicity [138]. These vaccines induce antibody production and offer comparatively strong safety profiles, with no risk of replication or disease transmission to the host [139]. This makes them suitable for mass administration in intensive broiler production systems [140]. For example, inactivated bacterial vaccines are widely used against organisms like *Pasteurella multocida* (Fowl cholera) with minimal concerns about environmental persistence [141]. As a major drawback, these vaccines can compromise the key surface epitopes and membrane proteins. As a result, the immune system induces the production of specific antibodies and other defenses against the compromised or altered structures. These antibodies fail to detect the native epitopes of the corresponding live pathogen. This epitope mismatch often leads to reduced vaccine efficacy against variable pathogens, the production of non-protective antibodies, loss of epitope conformation, and the frequent need for booster doses or adjuvants [142,143]. Thus, killed, or inactivated vaccines represent a safer but potentially less efficient option, particularly for complex, polymicrobial diseases such as BCO-associated lameness [32].

Live attenuated vaccines are produced by using living but weakened (avirulent or attenuated) microorganisms that can replicate within the host body without causing a clinical disease [144]. With a single dose, live attenuated vaccines can mimic natural infection and induce robust cell-mediated immunity, while enhancing long-lasting protection against bacterial pathogens [145]. Mass administration of live attenuated vaccines is cost-effective and aligns with broiler production cycles [146]. This platform has demonstrated effective, sustained, and strong immune responses in poultry models. For instance, the live attenuated *Salmonella typhimurium* vaccine has offered long-term efficacy against *Salmonella* infection in layer hens [147]. In addition, these vaccines have shown positive outcomes in reducing shedding, colonization, and potential cross-protection against bacterial pathogens such as *Salmonella* and *E. coli* [148,149,150]. However, there are several potential limitations of live attenuated vaccines, including reversion to virulent form, especially in immunocompromised birds, transient immunosuppression leading to secondary infections, shedding of vaccine strains, possible instability, and storage concerns such as cold-chain dependency [151]. Due to these reasons, live attenuated vaccines necessitate careful evaluation of persistence and safety for treating poultry disorders such as BCO, where gut leakage facilitates bacterial translocation [152]. Live attenuated vaccines exhibit high potency and scope but warrant rigorous attenuation strategies and monitoring to balance efficacy with safety risks.

Recombinant vaccines are highly specific vaccines produced using genetic engineering techniques and contain targeted components of the pathogen’s genetic material [153]. These vaccines act specifically against the pathogen of concern without affecting other bacteria. Generally, these vaccines are safer than live vaccines and can induce both humoral and cellular immunity [154]. However, the resulting immunity may be more limited due to fewer epitopes than in whole-cell vaccines, potentially reducing cross-protection against variant strains of the pathogen [28]. Subunit bacterial vaccines are prepared by purifying and isolating specific antigens from pathogenic bacteria [83]. These antigens are used to induce an immune response in the host’s body. Application of a liposomal subunit vaccine has demonstrated promise in reducing *Campylobacter* gut colonization in broiler chickens [155]. Subunit vaccines offer high efficacy against bacterial infections; however, multiple doses or adjuvants are required to maintain long-term immunity, which can be impractical in the short broiler lifespan [83]. Bacteriophage-based vaccines leverage viruses, and they are highly effective at controlling bacterial infections because they infect and destroy specific bacterial pathogens [28,156,157]. Key drawbacks include complex manufacturing requirements, potential immune system interactions, and a lack of long-term regulatory and clinical trial data [28,156,157]. All these vaccine platforms may provide insufficient coverage without careful antigen selection and combination strategies, for polymicrobial infections such as BCO.

### 5.3. The Efficacy of Bacterial Vaccines—A Comparative Analysis

Each bacterial vaccine platform has its own strengths and limitations determined by immunological mechanisms, production strategies, and practical applications. Table 2 summarizes the key strengths and limitations of available bacterial vaccines. The efficacy of bacterial vaccines is determined by vaccine type, target pathogen, management practices in the production system, age, and health of the broiler [28]. To date, several studies have attempted to develop vaccines against *Staphylococcus* spp., *Clostridium* spp., *Salmonella* spp., and *E. coli* for poultry, using target-specific proteins [158,159,160,161,162,163]. It has been demonstrated that live whole-cell *S. aureus* vaccines successfully elicited effective immune responses against *Staphylococcus* infection in a murine model when administered intravenously [163,164,165]. This illustrates the importance of administering whole-cell vaccines to elicit a highly effective, long-lasting immune response. The use of killed whole-cell bacterial vaccines is an interesting alternative to minimize the incidence of BCO lameness in broiler chickens. Presently, no commercial vaccines are available to prevent the disease [30,166].

## 6. Electron Beam Technology—A Novel Approach to Enhance the Efficacy of Vaccines

### 6.1. Electron Beam (eBeam) Technology

**Figure 4:**
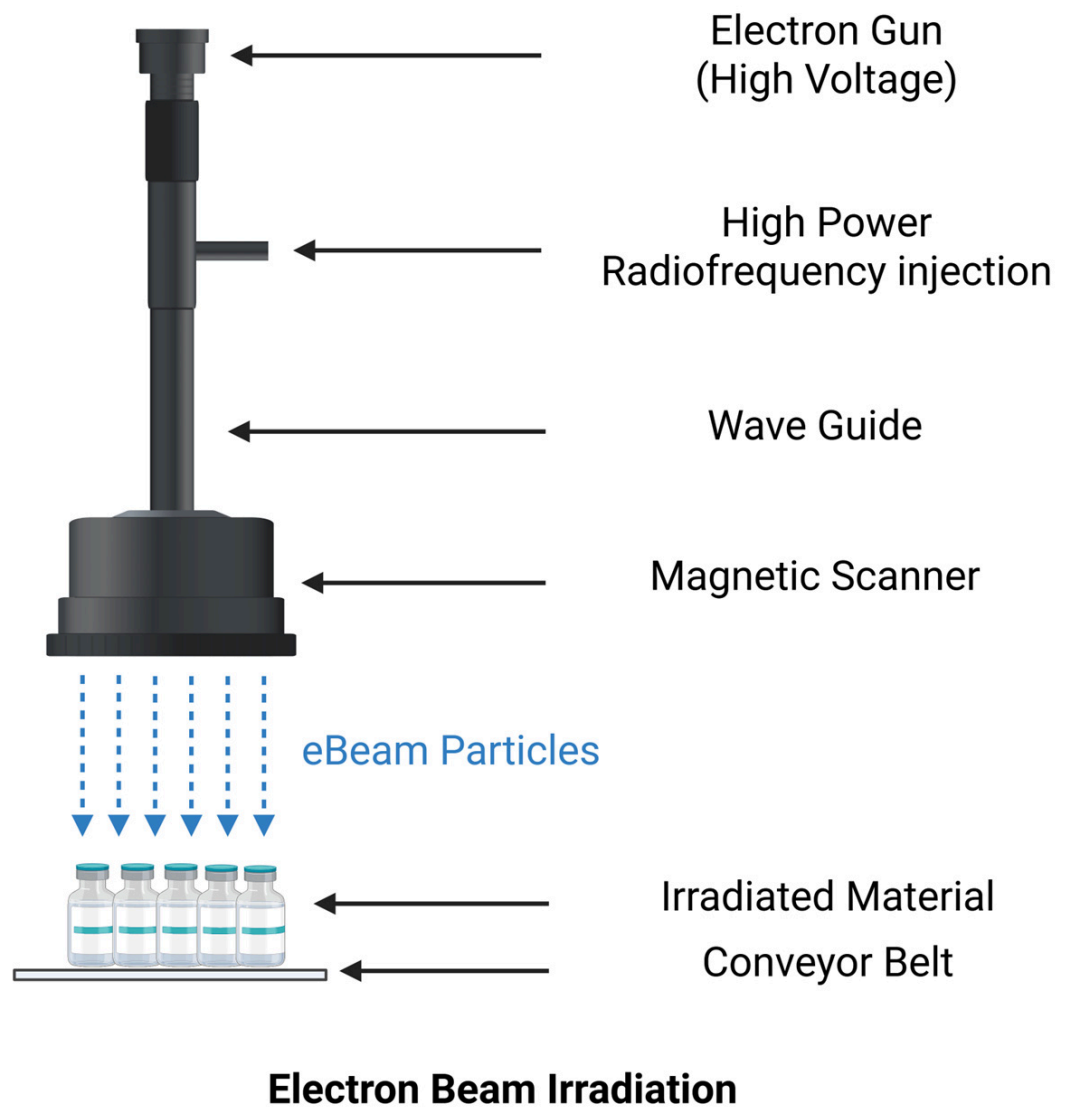
Schematic diagram of an eBeam accelerator. In this system, an electron gun operating at high voltage to accelerate electrons is followed by a high-power radiofrequency injection unit. A waveguide directs the electron beam, a magnetic scanner uniformly distributes the eBeam particles over the target material, and a conveyor belt ensures consistent electron exposure during irradiation. Created in BioRender. Rathnayake. (2026) (https://biorender.com/).

In recent years, electron beam technology (EBT) has been widely adopted across diverse fields, including food technology, agriculture, medicine, environmental applications, and industrial applications, due to its remarkable benefits, safety profile, and practical feasibility. Furthermore, eBeam is approved by the United States Food and Drug Administration (FDA) and the United States Department of Agriculture, Food Safety, and Inspection Service (USDA-FSIS) as an alternative approach to reducing foodborne pathogens [29,167,168]. Electron beams are produced using industrial equipment called electron accelerators. These accelerators produce high-energy electrons that can be used for numerous applications in diverse fields [169]. Electron accelerators consist of three primary components: the electron source, accelerator tube, and scan horn [170] (Figure 4). In these systems, electrons are emitted from a heated cathode through thermionic emission. The emitted electrons are further accelerated to high energies proportional to the accelerator voltage. The accelerated high-energy electrons are shaped into a beam using magnetic and electric fields. The products designed for eBeam treatment are packaged, loaded onto a conveyor system, and transferred through the eBeam zone. Doses of eBeam radiation are optimized to penetrate the product [171]. Electron beams are generally divided into three categories based on the energy levels: low-energy electron beam (<1 MeV; mega electron volts), medium-energy electron beam (1 MeV to 4 MeV), and high-energy electron beam (4 MeV to 10 MeV). The practical applications, interaction properties, and effectiveness in diverse industrial and medical scenarios are determined by these different energy levels. The low penetration power of EBT can be controlled by optimizing the size, dimensions, and specific density of the product prior to processing treatment.

### 6.2. Application of eBeam Technology in Vaccine Preparation

Vaccine development against microbial pathogens is a novel application of the EBT [31,32,85]. EBT inactivates microorganisms through a combination of direct and indirect effects, resulting in reproductive death and microbial inactivation [172]. This is a non-thermal process that breaks down vital cellular components, preventing reproduction and ensuring successful sterilization. The direct or primary effect of EBT is the nonspecific collision of highly energetic electrons with microbial cells, which ionizes macromolecules such as nucleic acids. The inactivation levels are determined by the absorbed dose, the state and age of the microbial cell, and the suspended matrix [29]. The principal target of ionizing radiation is the genetic materials of the cell, as they are the largest macromolecules within the cell. They form a major portion of cell components. Under optimized treatment doses and conditions, damage to proteins, lipids, and surface antigenic structures is minimal to none. When eBeam enters the microbial cell, it triggers nonspecific electron collisions, resulting in cleavage of phosphodiester bonds in the DNA backbone and hydrogen bonds between nitrogenous base pairs [173]. This leads to single- and double-stranded breaks in microbial DNA and the disintegration of the DNA double helix [174]. DNA disintegration inhibits DNA repair, DNA replication, and cell division, resulting in the microbe’s reproductive death. Genome size and radiation dose directly affect DNA disintegration [29,172,173,174,175]. Single-stranded breaks are partially repairable; however, extensive double-stranded breaks are considered irreparable. This direct damage or DNA disintegration is a relatively rapid process (10^−14^–10^−12^ s) and can efficiently generate inactivated cells that could be used as inactivated vaccines [29].

The indirect or secondary effects of EBT include the production of highly reactive radiolytic species (free radicals) such as aqueous electrons, hydrogen ions, hydrated protons, hydrogen peroxide, hydroxyl radicals, and hydrogen, due to the series of oxidation and reduction reactions of energetic electrons in eBeam with water molecules within the cell [29,170,173,176]. Another important secondary effect of EBT is the production of mutagenic lesions in the DNA. Highly reactive free radicals attack guanine (G), inducing the formation of 8-hydroxyguanine (8-oxoG or 8-oxo-7,8-dihydroguanine), also known as GO lesions [174,177]. The GO lesions are highly stable products of oxidative DNA damage, in which 8-hydroxyguanine pairs with adenine (A) rather than cytosine (C) during DNA replication, causing mutagenic G:C → T:A transversions [178]. Most importantly, EBT inactivates pathogens by inducing DNA damage while preserving the integrity of the cell membrane and leaving the antigenic surface epitopes intact [29,31]. Several studies demonstrated the preservation of structural integrity using fluorescence and electron microscopy [29,31,85,179]. This approach provides a safer and more effective alternative to conventional chemical inactivation methods for vaccine development under well-optimized conditions.

## 7. Electron Beam Inactivated Vaccines

### 7.1. eBeam-Inactivated Vaccines for Bacterial Diseases in Chickens

Several studies have evaluated the use of EBT to enhance vaccine efficacy against bacterial diseases in chickens. Kogut et al. (2012) [180] investigated a novel approach to administering a high-energy (10 MeV) eBeam-treated (2.5 kGy) *Salmonella enterica* serovar Typhimurium (ST) vaccine as a potential immune modulator to activate the innate immune response against intestinal colonization of *Salmonella* in neonatal chickens. In ovo vaccination of ST significantly reduced cecal colonization in the vaccinated group (0.95 ± 0.23 log_10_ CFU/g) compared with the nonvaccinated group (3.48 ± 0.59 log_10_ CFU/g). In this experiment, 18-day-old chicken embryos were treated with a sham-injected, no-challenge (negative control), a sham-injected, challenged (infected control), a CpG oligodeoxynucleotides (CpG-ODN)-injected, challenged positive control, and an eBeam ST-injected, challenged group to evaluate and compare heterophil function using in vitro assays for oxidative burst and degranulation. Heterophil function of the CpG-ODN-injected birds, and eBeam ST-injected birds demonstrated a significant increase (*p* < 0.05) in both the oxidative response and degranulation when compared to other treatment groups. However, there is no significant difference in heterophil functions between the two groups. In both the CpG-ODN- and eBeam ST-treated groups, cecal ST colonization was significantly reduced (*p* < 0.05) compared with the nonvaccinated control groups.

A study conducted by Jesudhasan et al., 2015 [31] provided the first evidence that EBT can be used for vaccine development by inactivating bacterial pathogens while preserving their cellular structure and integrity. This study demonstrated that the administration of eBeam-inactivated *Salmonella enterica* serovar Enteritidis (SE) cells can serve as an effective vaccine to control SE colonization in molting hens. During vaccine preparation, relatively high-titer SE cultures (10^8^ CFU/mL) were inactivated using an eBeam dose of 2.5 kGy. When examined under fluorescence microscopy, the eBeam-treated SE and nontreated SE cells stained with the live/dead bacterial viability stain revealed that most eBeam-treated SE cells were green (living) and only a few were red (dead), under both treatments. Transmission Electron Microscope (TEM) or Scanning Electron Microscope (SEM) images of the eBeam-treated and untreated SE cells showed no significant differences in membrane structure or integrity, and both samples exhibited micro-vesicles on the cell surface. When incubated overnight at 37 °C or for up to 4 weeks at room temperature in tryptic soy agar and tryptic soy broth, the eBeam-treated SE cells showed no growth. Vaccination of eBeam-killed SE cells into 50-week-old single-comb white leghorn hens (1 × 10^6^ CFU/mL, intramuscular) showed significant (*p* < 0.0001) SE-specific IgY antibody responses. Furthermore, vaccinated birds exhibited significantly lower SE colonization in the ceca (1.46 ± 0.39 log_10_ CFU/g) compared to nonvaccinated birds (5.32 ± 0.32 log_10_ CFU/g), along with lower SE colonization rates in ovaries, spleen, and liver. In both the vaccinated and unvaccinated birds, neither abnormal clinical signs nor morbidity were observed. These findings confirmed that eBeam-treated SE vaccines are immunogenic and provide protective efficacy against SE colonization in chickens.

Moreover, Jesudhasan et al. (2021) [85] reported that EBT can be used to generate effective vaccines against *Clostridium perfringens* (CP) infection in chickens. In this study, three different CP strains were inactivated using eBeam treatment (10 MeV, 18 kW) and then used as a killed vaccine to control CP colonization in broiler chickens. In ovo administration of eBeam CP into 18-day-old embryos showed that an effective eBeam dose of 10 kGy inactivated all three CP strains and did not affect cell membrane structure, integrity, metabolic activity, or surface epitopes, as confirmed by AlamarBlue assays, fluorescence, TEM, and SEM measurements. The immunization also induced a significant increase in serum IgY levels and a significant reduction (*p* < 0.0001) in intestinal colonization by CP strains during late challenge. In both vaccinated and unvaccinated birds, no significant clinical signs, weight loss, morbidity, or death were observed. This study highlights the efficacy of an eBeam vaccine, which combines the immunogenic potential of a live vaccine and the safety of a killed vaccine.

### 7.2. An Innovative Approach to Mitigate BCO Lameness in Broiler Chickens

Few recent studies have demonstrated that eBeam-killed *Staphylococcus* vaccines significantly reduced the incidence of BCO lameness in broiler chickens, and this novel knowledge provides critical insights for the development of the poultry industry [30,32]. A summary of the key findings from major studies evaluating eBeam-killed *Staphylococcus* vaccines is presented in Table 3.

Assumpcao et al., 2024 [30] represent a landmark contribution in the poultry industry as the first to report the successful development of eBeam-killed *Staphylococcus* vaccine against BCO. During eBeam-killed vaccine preparation, bacterial strains (*S. aureus* and *S. agnetis*) were inactivated using high-energy (10 MeV, 15 kW) eBeam radiation at a dose of 10 kGy. Fluorescence microscopy revealed that the eBeam-treated bacteria were effectively inactivated while maintaining cell membrane integrity and preserving antigenic epitopes. The eBeam-treated and formalin-killed vaccines were administered in ovo to broilers, along with a sham vaccine, to mimic commercial hatchery practices. A total of 210 one-day-old chicks were assigned to three experimental groups (eBeam-treated, formalin-killed, and control), with two replicate pens per treatment and 35 birds per pen. The vaccine efficacies were evaluated in a controlled lameness drinking-water challenge model (*S. agnetis*, 10^4^ CFU/mL) on days 20 and 21 post-hatch. The experimental data showed that the eBeam-vaccinated group exhibited approximately a 50% reduction in clinical lameness incidence compared to the formalin-killed (3%) and control groups. Furthermore, odds ratio analysis showed a significantly lower probability of developing lameness in the eBeam-treated group (*p* = 0.0008), but similar probabilities between the formalin and control groups (*p* > 0.05). The contrast analysis showed that the odds of lameness in the eBeam treatment and formalin groups were 18.5% and 88.2% of the control group’s odds, respectively. In this study, ELISA was performed to quantify antibody titers in serum. The eBeam-treated group had significantly higher IgA titers in serum than the control group. These results indicated a significantly higher IgA secretion in the mucosa, which plays a critical role in neutralizing pathogens before they can penetrate the mucosa and translocate into the bloodstream. Above findings demonstrated that the eBeam-killed *Staphylococcus* vaccine can induce an efficient and effective IgA-mediated immune response and significantly reduce BCO lameness in broiler chickens compared to the formalin-treated vaccine.

Perera et al. (2025) [32] utilized a modified version of the eBeam-inactivated *Staphylococcus* vaccine against BCO lameness in broiler chickens, with an optimized dose and multiple *Staphylococcus* strains frequently isolated from BCO lesions. In this experiment, birds were challenged with the natural intestinal and environmental microbiota using an aerosolized BCO challenge model [182] to mimic a real-world setting closely. A high-energy (10 MeV, 15 kW) eBeam radiation at a further optimized dose of 8 kGy was used to completely inactivate the bacterial strains (*S. agnetis*, *S. aureus*, *S. lentus*, and *S. cohnii*) while preserving bacterial cell viability, metabolic activity, and membrane integrity. In contrast, the viability, metabolic activity, and membrane integrity of formalin-treated *Staphylococcus* cells were significantly lower than those of the eBeam-treated cells. These data were confirmed by microbial cell viability and fluorescence microscopy assays, respectively. The vaccines were administrated in ovo, and five treatment groups were utilized as follows: (1) birds raised on a wire floor as the infection source and vaccinated with a sham (WF), (2) birds vaccinated with eBeam-inactivated vaccine (eB), (3) Birds vaccinated with formalin-inactivated vaccine (FK), (4) birds vaccinated with a combination of the eBeam and formalin-inactivated vaccines (EF), (5) birds vaccinated with a sham as a control group (C). Vaccination of eBeam-inactivated multi-strain *Staphylococcus* cells demonstrated a highly significant reduction (50.94%, *p* < 0.05) in daily cumulative BCO lameness by day 56 (32.22% lameness incidence) compared to the control group (67.5% lameness incidence). At the same time, formalin-inactivated and combination vaccines did not significantly reduce lameness. Unlike in other treatment groups, *Staphylococcus* spp. were not isolated from lame birds in the eBeam-treated group, possibly due to the target-specific protection conferred by the eBeam vaccine. The eBeam-treated and combination vaccine groups demonstrated higher tracheal mucosal IgA levels, while the formalin-treated and sham-vaccinated groups showed lower levels, indicating effective protection against pathogens transmitted via the respiratory tract conferred by the eBeam and combination vaccines. Collectively, this evidence confirmed that an eBeam-inactivated *Staphylococcus* vaccine induces robust protection against BCO lameness by preserving critical surface epitopes, eliciting rapid mucosal IgA responses, and providing more stable immune responses against infection.

Anthney et al. (2025) [181] emphasized the fundamental role of eBeam-killed *Staphylococcus* vaccine (the same vaccine used in the previous study) in mitigating the incidence of BCO lameness while preserving the immunogenicity and safety. In this experiment, the multivalent eBeam-vaccine was administered alone and in combination with two probiotics (GALLIPRO^®^ Hatch [GH] and GALLIPRO^®^ Fit [GF]), using the aerosol transmission challenge model. The eBeam vaccine was administrated in ovo into the embryonic cavity (day 18), GH probiotic was administrated (*Enterococcus faecium* strain −2.0 × 10^9^ CFU per chick) using an in-house static spraying system at hatch (day 0), and GF probiotic (*Bacillus subtilis* strains—5.0 × 10^8^ CFU or 1.0 × 10^9^ CFU per bird) was supplied in powdered form and diluted with drinking water consistently throughout the study period. In this study, five treatment designs were used: (1) Positive control—PC (Infection source—birds raised on wire floor), (2) Negative control—NC, (3) GH/GF—T1, (4) eBeam vaccine—T2, and (5) GH/GF and eBeam vaccine combination—T3. Daily cumulative lameness incidence varied significantly across treatment groups. The highest cumulative lameness incidence was observed in the PC and NC groups, at 83% and 71%, respectively. In contrast, treatment groups T1, T2, and T3 demonstrated significantly reduced cumulative lameness rates at 43.7%, 40.3%, and 40.7%, respectively (*p* < 0.05). Moreover, treatment groups experienced a more gradual increase, whereas the PC and NC groups showed sharper increases in the cumulative lameness rate. Additionally, in all treatment groups, severity level and frequency of tibial lesions gradually decreased, but no clear pattern was observed for femoral lesions. These findings highlight the strong efficacy of the eBeam-inactivated *Staphylococcus* vaccine as an antibiotic-free strategy, demonstrating a significant reduction in BCO lameness incidence compared with the probiotic-only-treated group.

## 8. Discussion

BCO lameness remains one of the most economically and welfare-significant infectious musculoskeletal disorders in modern broiler production. To date, BCO incidences were partially controlled by non-vaccine control strategies [18,22,65,104,114]. The development and spread of AMR has reduced the efficacy and long-term viability of antimicrobial treatment [78,81,82,114,115], highlighting the critical need for alternative strategies for BCO prevention and control [29]. Vaccination against bacterial diseases is considered one of the most promising and targeted immunological approaches, as it prepares the host immune system to eliminate pathogens before actual infection [28].

### Critical Perspectives on eBeam Technology

Among other vaccine platforms, bacterial inactivation using EBT represents a significant advancement and novel approach in poultry production [31,174]. Recently, eBeam-inactivated vaccines have gained attention for their ability to achieve reproductive inactivation through DNA damage while preserving bacterial cell viability, membrane integrity, native surface epitopes, and metabolic activity, compared to traditional formalin-inactivated vaccines [29,30]. Several studies from controlled models have demonstrated the efficacy of in ovo administration of eBeam-inactivated multivalent *Staphylococcus* vaccines, achieving a significant reduction in BCO lameness incidences (approximately 40–50%) and decreased bacterial colonization in bone lesions [30,32].

Despite the promising results of EBT in vaccine production, a critical evaluation reveals important limitations and challenges. Due to the novelty of this approach, most of the published data in this review were derived from a relatively small number of studies conducted primarily in our lab, which raises concerns about the generalizability and reproducibility of data. Another notable concern is that most experiments have been conducted primarily using an aerosol transmission model on wire flooring, which may not accurately reflect the environmental variability in large commercial broiler production houses. Studies conducted to date have primarily focused on *Staphylococcus* spp., leaving a need for multivalent vaccines that simultaneously target *E. coli* and *Enterococcus* spp. underdeveloped [29,30]. Transitioning eBeam-inactivated vaccines from experimental trials to real-world settings depicts several technical, economic, and practical challenges. Generally, a precise dose optimization is required to achieve complete genetic inactivation of bacteria without impairing immunogenicity [32,183]. Supra-optimal doses may compromise epitope quality by causing oxidative damage to proteins and lipids, while sub-optimal doses may lead to incomplete genetic inactivation and potential safety concerns [29,32]. EBT requires significantly higher capital investment in accelerators, shielding, and trained personnel; thus, economic analyses are essential to evaluate economic return. Sustainability assessments should be conducted with environmental considerations in mind, such as EBT’s energy consumption. Additionally, long-term vaccine trials across multiple geographic regions should be conducted under diverse commercial production conditions, including varying stocking densities, nutritional quality, housing conditions, and genetic lines, to determine efficacy and validity [30]. Moreover, accurate and comprehensive studies on manufacturing scalability, regulatory approval pathways, and economic cost-benefit analyses should be performed prior to the widespread industry application of EBT for commercial broiler vaccines. Table 4 summarizes key advantages and disadvantages of EBT.

## 9. Future Directions and Conclusions

Future research should focus on several high priority areas with strong research potential to advance EBT for the control of BCO lameness. First, multiple research groups across different geographical areas should conduct independent studies under variable production systems, to validate the performance and efficacy of eBeam-treated vaccines. Second, development of broader multivalent eBeam vaccines that target the complete spectrum of BCO lameness-associated pathogens, including *E. coli* and *Enterococcus* spp., could enhance the vaccine efficacy and provide cross-protection against emerging strains [29,30]. Third, integrated approaches such as eBeam vaccination with optimized probiotics, precision nutrition, microbiome intervention, or genetic markers for skeletal integrity, may offer positive synergistic effects on BCO control [181]. Fourth, advanced drug delivery systems particularly nanoparticles/nanocarriers, targeted delivery mechanisms, stimuli-responsive systems and slow-release formulations could enhance the immune responses, provide long-lasting protection, improve long-life cycle performance, and improve vaccine efficacy [189,190].

In conclusion, eBeam-inactivated whole-cell vaccines offer a scientifically grounded and innovative approach to mitigating BCO-associated lameness in broiler chickens. Preliminary studies under experimental conditions, have yielded significant reductions in clinical lameness incidences and bacterial colonization in growth plates. Nevertheless, due to current limitations and challenges; the efficacy and safety of eBeam-treated vaccines should be further validated independently. With continued rigorous research, and strategic integration with genetic improvements, nutritional interventions, and environmental optimization, EBT can potentially contribute to mitigating BCO-associated lameness and to more sustainable, welfare-oriented, and antibiotic-free broiler production systems.

## Figures and Tables

**Table 1:** Non-vaccine control and mitigatory strategies for BCO lameness in broiler chickens, including reported efficacy and limitations.

Control and Mitigatory Strategy	Specific Interventions	Reported Efficacy on BCO Lameness Incidence	Major Limitations	References
Genetic selection and breeding approaches	Selection for tibial breaking strength, cortical thickness, bone mineral density, and gait score; genomic selection for leg health traits	Moderate reduction in musculoskeletal abnormalities such as angular deformities, tibial dyschondroplasia, and lameness	Strong link to rapid growth and production traits, slow genetic progress, and complexity, limited standalone efficacy	[4,6,59,60,61,62,63,64]
Effective management of broiler production environment	Optimized lighting programs, adequate ventilation, thermal regulation	Moderate reduction in leg abnormalities, improved bone strength and cortical thickness	Limited direct effect on bacterial translocation, high infrastructure cost	[61,65,66,67,68,69,70]
Nutritional Interventions	Probiotics, trace minerals, balanced Ca:P ratio, Vitamin D3 supplementation, amino acids	Significant reduction in lameness incidence, improvement in bone mineralization and reduced lameness	Efficacy is variable depending on microbial strain in probiotics, dose, and challenge level, short duration of effect, over-supplementation risk	[47,71,72,73,74,75,76,77]
Use of antimicrobial agents	Tetracyclines, fluoroquinolones, sulfonamides, aminoglycosides, colistin	Significant short-term reduction in BCO incidence	Rapid development of antimicrobial resistance, not sustainable	[24,28,78,79,80,81,82,83,84,85]

**Table 2:** Key strengths and limitations of bacterial vaccines used in poultry production.

Vaccine Type	Strengths	Limitations	References
Killed or inactivated	Reliably strong safety profiles, easier to produce; no risk of replication or disease caution; suitable for mass administration	Alter key surface epitopes and surface proteins; requires booster shots or adjuvants	[140,141,142,143]
Live attenuated	Mimics natural infection; induces robust immune responses; cost-effective, long-lasting protection; cross protection against variable strains	Reversion to virulent form, leading to secondary infections; shedding of vaccine strains; possible instability; storage concerns	[144,145,146,148,149,150,151]
Recombinant	Highly specific and strong safety profiles; induce both humoral and cellular immunity	Limited immunity due to fewer epitopes; weak cross-protection; requires booster shots or adjuvants	[28,154]
Subunit	High efficacy against bacterial infections	Requires multiple doses or adjuvants to maintain long-term immunity	[83,155]
Bacteriophage-based	Highly effective, infect and destroy specific bacterial pathogens	Manufacturing complexity; lack of long-term regulatory and clinical trial data	[156,157]

**Table 3:** Studies on eBeam-killed *Staphylococcus* vaccines against bacterial chondronecrosis with osteomyelitis-induced lameness in broiler chickens.

Study	Vaccine Type and Administration	eBeam Dose (kGy)	Challenge Model	Lameness Reduction (%)	Key Immune Findings	Main Conclusion
Assumpcao et al. (2024) [30]	Multivalent eBeam-killed (*S. aureus* and *S. agnetis*)—in ovo	10	Drinking water oral challenge	50 (*p* < 0.05)	Significantly higher serum anti-*Staphylococcus* IgA at day 33, indicating strong mucosal immunity	First report of eBeam-killed *Staphylococcus* vaccine, markedly superior to the formalin-killed version with clear protection via preserved surface epitopes
Perera et al. (2025) [32]	Multivalent eBeam-killed (*S. aureus*, *S. agnetis*, *S. lentus* and *S. cohnii*)—in ovo	8 (Optimized)	Wire floor aerosol transmission model	50.94 (*p* < 0.05)	Highest tracheal mucosal IgA at day 16, suggesting early mucosal protection. Completely prevented *Staphylococcus* colonization in BCO lesions	Optimized lower eBeam dose achieved complete bacterial inactivation while retaining high immunogenicity
Anthney et al. (2025) [181]	Multivalent eBeam vaccine	8	Wire floor aerosol transmission model	43.2 (*p* < 0.05)	-	Multivalent eBeam vaccine alone provided robust and consistent lameness reduction comparable to combination therapy

**Table 4:** Key advantages and disadvantages of eBeam technology.

Key Aspect	Advantages	Disadvantages	References
Bacterial inactivation	Irreversible DNA damage; no risk of replication	Inactivation efficacies depend on dose optimization	[30,183]
Antigen preservation	Preserve confirmational epitopes and antigenic proteins compared to chemically inactivated vaccines	Supra-optimal doses may alter epitope quality	[29,31,32]
Penetration depth	Suitable for thin materials and surface sterilization	Not suitable for thick or high-density materials	[184,185,186]
Scalability and cost	Rapid non thermal process	High infrastructure costs; specialized facilities required	[185,187]
Environmental impact	No chemical residue; minimal waste generation	High electricity demand	[187,188]

## Data Availability

No new data were created or analyzed in this study. Data sharing is not applicable to this article.
